# Socio-Demographic Factors Associated with Rural Residents’ Dietary Diversity and Dietary Pattern: A Cross-Sectional Study in Pingnan, China

**DOI:** 10.3390/nu15132955

**Published:** 2023-06-29

**Authors:** Lingling Zhang, Huajing Chang, Yating Chen, Wenqian Ruan, Longhua Cai, Fang Song, Xiaojun Liu

**Affiliations:** 1 Department of Epidemiology and Health Statistics, School of Public Health, Fujian Medical University, Fuzhou 350122, China; 2210220176@fjmu.edu.cn (L.Z.); huajing_chang@fjmu.edu.cn (H.C.); rwq@fjmu.edu.cn (W.R.); 2 Department of Health Management, School of Health Management, Fujian Medical University, Fuzhou 350122, China; yating_tina@fjmu.edu.cn (Y.C.); clh6389@stu.fjmu.edu.cn (L.C.); 3 Editorial Department of Medicine and Society, Tongji Medical College, Huazhong University of Science and Technology, Wuhan 430030, China; 13419521042@163.com

**Keywords:** dietary diversity, dietary pattern, socio-demographic factors, rural residents

## Abstract

There is limited evidence regarding the factors correlated with dietary diversity (DD) and dietary pattern (DP) in rural residents of China. This study aims to identify the DD and DP of rural residents and their association with socio-demographic factors. A cross-sectional survey was conducted in Pingnan, China. The Food Frequency Questionnaire (FFQ) was applied to evaluate dietary intake. Latent class analysis (LCA) was used to identify patterns of six food varieties, including vegetables–fruits, red meat, aquatic products, eggs, milk, and beans–nuts. Generalized linear models and multiple logistic regression models were used to determine factors associated with the DD and DP. Three DPs were detected by LCA, namely “healthy” DP (47.94%), “traditional” DP (33.94%), and “meat/animal protein” DP (18.11%). Females exhibited lower DD (β = −0.23, *p* = 0.003) and were more likely to adhere to “traditional” DP (OR = 1.46, *p* = 0.039) and “meat/animal protein” DP (OR = 2.02, *p* < 0.001). Higher educational levels and annual household income (AHI) were positively associated with higher DD (*p* < 0.05) and less likely to have “traditional” DP and “meat/animal protein” DP (*p* < 0.05). Non-obese people exhibited higher DD (β = 0.15, *p* = 0.020) and were less likely to have “meat/animal protein” DP (OR = 0.59, *p* = 0.001). Our study reveals that females, those with lower educational levels and AHI, and obese people are more likely to have a lower DD and are more likely to adhere to “traditional” DP and “meat/animal protein” DP. The local, regional, and even national performance of specific diet-related health promotion measures and interventions must target these vulnerable populations to improve a healthier DD and DP.

## 1. Introduction

Dietary intake is well known as a significant determinant of health [1], and improving dietary patterns are likely to improve population morbidity and mortality [2]. According to previous work, dietary intake is a complicated behavior that cannot be reduced to consuming a single type of food [3]. Indeed, the types of food and their interactions hinder the investigation of the links between specific foods and diseases [4]. For this reason, the study needs to shift to dietary diversity (DD) and dietary pattern (DP) analysis to measure the multidimensional features of food intake. DD is known as the number of different food varieties consumed over a given reference period [5], and has been recommended as an indicator for evaluating the composition of the diet [6]. High DD scores have been strongly associated with better physical performance, lower mortality, and higher quality of life [7,8]. National and international dietary standards have largely acknowledged the importance and value of DD [9]. Similarly, DPs reflect the interrelations among the different food varieties and provide a more accurate depiction of dietary behavior [4]. It fosters further research regarding single foods and nutritional guidelines due to providing the context for identifying specific food groups that are either protective or harmful [10]. A systematic review has shown that following healthier dietary patterns (DPs), such as the Mediterranean DP, can reduce the risk of mortality and non-fatal myocardial infarction in people [11]. However, socio-demographic factors have been shown to significantly influence food choices [12,13]. Therefore, it is crucial to identify determinants of DD and DP in the population and to explore whether they contribute to previously described socio-demographic differences in disease prevalence.

Several factors associated with DD and DP have been previously reported [14,15,16]. Previous studies have found significant associations between sex and DD or DP. Specifically, males are usually related to an unhealthy pattern, while overweight or obese females are more likely to experience food cravings and consume more foods [14,17]. Moreover, older adults typically make positive decisions concerning their nutrition and health and tend toward a healthier diet than younger people [18,19]. Additionally, socioeconomic factors such as education level, financial situation, and knowledge of a healthy diet also significantly influence DD and DP, mainly due to the lower intake of proteins, fruits, and vegetables [16,17,20]. Healthy foods, including protein-rich foods, are more expensive than foods high in saturated fat or carbohydrates [21]. From a public health perspective, understanding the related factors of high (low) DD and the (un) healthier DP for different groups of individuals will allow for improved diet policy and aid in health resource allocation [4]. Although relevant research is already assessing risk factors for DD and DP, some of these studies have been confined to susceptible groups for more specificity. Primarily, it is vital to acknowledge that mainland China has a large number of farmers, and rural areas are the most vulnerable areas and require special attention [22]. However, there is limited evidence regarding the factors correlated with DD and DP in rural residents.

With the rapid economic and social development, Chinese rural residents have changed the single-food diet in the past and are constantly moving towards a healthy diet. Nevertheless, a small number of foods consumed and an unreasonable single-food DP remain a big concern in many rural areas, particularly in underdeveloped areas [23]. Pingnan is an impoverished county in Fujian Province’s inland and mountainous regions. The seventh national census, completed in 2021, recorded a number of 139,815 inhabitants living in Pingnan [24]. According to the Statistical Bulletin on National Economic and Social Development of Pingnan in 2022, the annual disposable income per capita was CNY 26,416 (equivalent to USD 3720 using an exchange rate of USD to CNY 1 to 7.10) [25]. Under the background of comprehensively promoting rural revitalization to achieve more effective and comprehensive health poverty alleviation strategies, the Adult Chronic Disease and Nutrition Surveillance were conducted in Pingnan by the Provincial Center for Disease Control and Prevention (CDC). Using data from this surveillance, this study aims to discover the DD and DP with the socio-demographic correlates for rural residents. The study’s findings may provide the necessary scientific basis for the local and regional relevant government departments to develop or adjust more effective measures and strategies, as well as good health diet programs, to improve DD and DP in rural residents by targeting specific populations.

## 2. Materials and Methods

### 2.1. Study Design and Samples

The cross-sectional data were derived from the Adult Chronic Disease and Nutrition Surveillance in Pingnan, China, between June and August 2022. The questionnaire used in the survey was based on previous Nutrition and Health Surveys conducted in China, which can increase the reliability of the data. Detailed information about this survey was given in a report from China CDC [26]. This survey adopted a conversational design and a multistage systematic clustered random sampling design to select subjects with a good representative sample. In the first stage, the number of inhabitants in different towns/villages were thoroughly investigated before sampling. According to the latest Pingnan census data, Pingnan is divided into 11 towns/villages. One of the towns was divided into six sampling units due to the large population to match with other towns/villages. A total of 16 towns/villages were included in the first-stage sampling, and the population distribution of each sampling unit was relatively balanced. The Probability Proportionate to Size (PPS) [26,27] sampling was adopted to select six towns/villages from 16 towns/villages. Then, three villages were randomly selected from each selected township by PPS, and a total of 18 villages were finally selected. If the population was too small, two or more villages situated next to each other were merged into one sampling unit. Next, each selected village was divided into several groups of villagers/residents according to the scale of about 60 households. Two groups of villagers/residents were randomly selected from each village by PPS, and a total of 36 groups of villagers/residents were used for this study. In the last stage, one target respondent aged 18 and over was sampled from 50 households per resident group using the Kish selection table method. There are ten households reserved for replacement. Overall, 1800 participants were included in the survey.

Inclusion criteria: participants who: (1) were aged 18 and over; (2) were residents or lived locally for over six months; (3) were conscious, without psychiatric problems/disorders; (4) were informed of the purpose of the study and willing to cooperate; (5) were able to complete the survey. The exclusion criteria were those unable to complete the questionnaire due to critical diseases or poor compliance and non-cooperation.

### 2.2. Data Collection

In our study, well-trained investigators fluent in the local dialect conducted face-to-face interviews using a standardized paper questionnaire to collect information on demographic characteristics and dietary intake. The demographic information includes gender, age, marital status, educational level, annual household income (AHI), smoking, drinking, waist circumference, hypertension, and diabetes. Age was further divided into three groups: 18–44 (younger people), 45–59 (middle-aged people), and over 60 (older adults). Marital status was grouped into married (married/cohabitating) and others (unmarried/widowed/divorced/separated). Educational level was classified into five groups: below primary school, primary school, junior high school, senior high school, and junior college or above. AHI was categorized into four groups: <12,000 yuan, ≥12,000 yuan and <19,999 yuan, ≥20,000 yuan and <59,999 yuan, and ≥60,000 yuan. Smoking was classified as current and non-current smoking (including never smoked and already quit smoke). Drinking was categorized as drinking (including drinking within 30 days and before 30 days) and non-drinking. Obesity assessed by abdominal obesity was defined as yes if a waist circumference of ≥90 cm for men and ≥85 cm for women in physical examination. Hypertension was defined as yes if answered as diagnosed with high blood pressure by a doctor in township health centers or community service centers, or medical institutions above the level. Diabetes was defined as yes if answered as diagnosed with diabetes by a doctor in township health centers or community service centers, or medical institutions above the level.

A variety of dietary intake in the past 12 months was surveyed by uniformly trained investigators using the Food Frequency Questionnaire (FFQ) to evaluate the dietary structure. FFQ are available in previous Adult Chronic Disease and Nutrition Surveillance Surveys with good reliability and validity [26,28]. Our study selected six food varieties: vegetables–fruits, red meat, aquatic products, eggs, milk, and beans–nuts. There are options for intake, yes or no, to ask about food frequency. The choice for yes was four optional responses “daily”, “weekly”, “monthly”, and “yearly.” Vegetables–fruits were defined as sufficient if answered daily/weekly and were coded as 1. If they responded to others, they were coded as 0. Red meat was defined as sufficient if answered daily and was coded as 1. If they responded to others, there were coded as 0. Aquatic products, eggs, milk, and beans–nuts were defined as sufficient if answered daily/weekly and were coded as 1. If they responded to others, they were coded as 0. Finally, six food varieties were formed. DD was calculated by adding together these six food varieties. The DD ranged from zero (none of the six selected food varieties occurred) to six (all six selected food varieties occurred). A higher DD means more diversity and a wider variety of food intake.

### 2.3. Statistical Analysis

Data analysis consisted of four steps. Firstly, a descriptive analysis of the demographic characteristics, six selected food varieties, and DD was conducted with frequencies and proportions. Secondly, a Latent Class Analysis (LCA) was used in six food varieties to identify the DP of rural residents in Pingnan. Three DPs were identified. A high score indicates a high probability of sufficient food variety intake. Thirdly, the Chi-square test was performed to compare the demographic characteristics across the DD and DPs. Afterwards, generalized linear models were used to assess factors associated with the DD, and the coefficient (β) with associated 95% confidence interval (CI) and *p*-values were presented in the model. Finally, multiple logistic regression analysis was used to identify the influencing factors of different DPs, and odds ratios (ORs) as well as 95% confidence intervals (CIs) and *p*-values were calculated. The database was established and double-entered independently through Epidata 3.1 and analyzed in the Statistical Package for the Social Sciences (SPSS) version 25.0 (SPSS Inc., Chicago, IL, USA) and Mplus version 8.3, with a significance level of 0.05.

LCA is a methodological approach that explains population heterogeneity in the data by identifying underlying subgroups of individuals, thus allowing the examination of different DPs while dealing with the diverse nature of the population [29]. For the model evaluation, five model fit indexes were adopted: the Akaike Information Criterion (AIC) [30], the Bayesian Information Criterion (BIC) [31], the sample size adjusted Bayesian Information Criterion (ssaBIC) [32], Lo-Mendell-Rubin (LMR), Bootstrapped Likelihood Ratio Test (BLRT), and Entropy (higher value is preferred) [33]. For AIC, BIC, and ssaBIC, the lowest absolute values suggest an excellent model class [34]. With LMR and BLRT, a significant *p*-value indicates that the model is superior to the model with one less class [29]. Nonetheless, the final choice was based on the investigator’s assessment of interpretable results [35]. This study chose the 3-class because of the lower AIC, BIC, and ssaBIC, higher entropy, and LMR-LRT and BLRT < 0.001.

## 3. Results

### 3.1. General Demographic Characteristics of the Study Participants

The general demographic characteristics of the study participants are shown in Table 1. A total of 1800 rural residents were included in the study, among whom 888 were males (49.33%), and 912 were females (50.67%). Participants were divided into age groups, 18–44 years, 45–59 years, and 60 years or older, accounting for 18.61%, 41.44%, and 39.95% of the total sample, respectively. Most respondents were married (86.44%) with a primary school education or lower (59.05%). Participants’ AHI ranged from 20,000 to 59,999 yuan (43.61%) and 60,000 yuan or more (23.83%), accounting for most of the sample. Moreover, 29.06% of respondents reported smoking, 27.50% said drinking, 27.61% had obesity, 33.89% had hypertension, and 17.28% had diabetes.

### 3.2. The Six Food Varieties and Dietary Diversity

**Figure 1:**
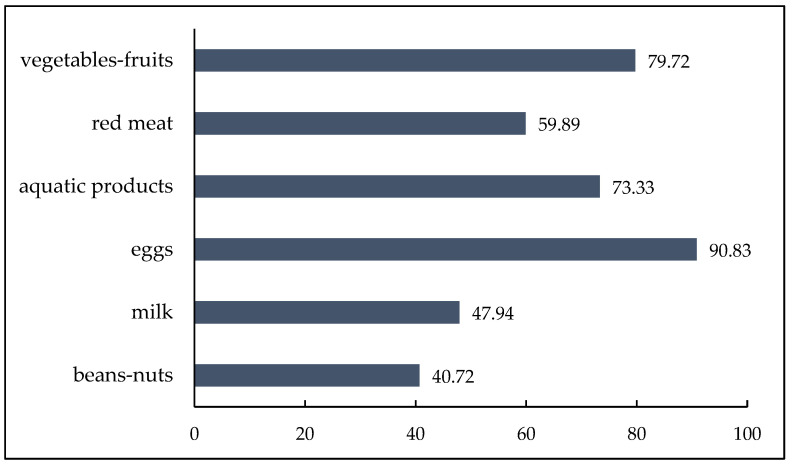
The percentage of six food varieties (%).

**Figure 2:**
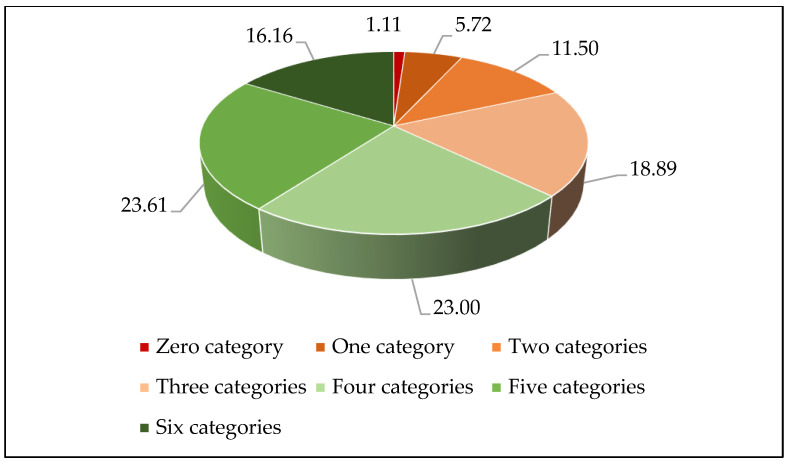
The constitution ratio for the dietary diversity (%). Note: Percentages may not add up to 100% due to rounding.

Figure 1 shows the characteristics of percentages for food varieties. Of the six food varieties, participants who intake sufficient eggs, vegetables–fruits, aquatic products, and red meat presented high rates, with 90.83%, 79.72%, 73.33%, and 59.89%, respectively. In contrast, those who intake sufficient milk and beans–nuts presented low percentages, with 47.94% and 40.72%. Figure 2 illustrates the constitution ratio of the DD. Among those respondents, 18,55% had two or lower food varieties, 18.89% had three food varieties, 23.00% were four food varieties, 23.61% were five food varieties, and 16.16% reported six food varieties.

### 3.3. Latent Class Analysis of Dietary Patterns

**Figure 3:**
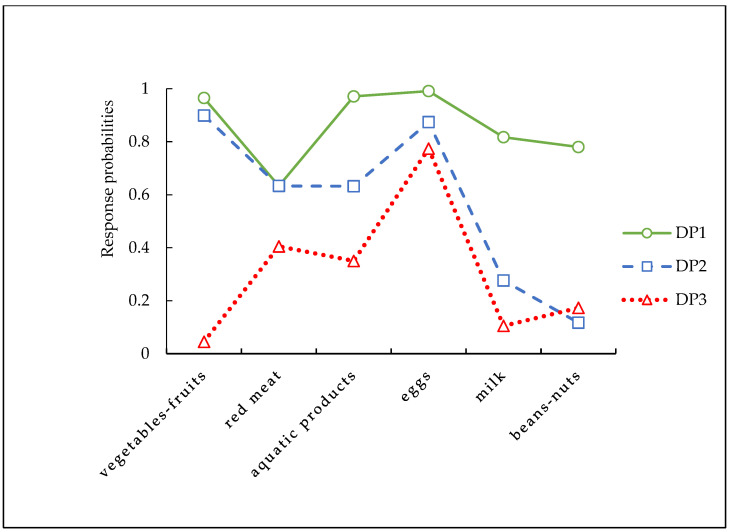
Estimated class-specific response probabilities for six food varieties. Note: A high score indicates a high probability of a sufficient food variety intake. Abbreviation: DP 1 = “healthy” dietary pattern, DP 2 = “traditional” dietary pattern, DP 3 = “meat/animal protein” dietary pattern.

**Figure 4:**
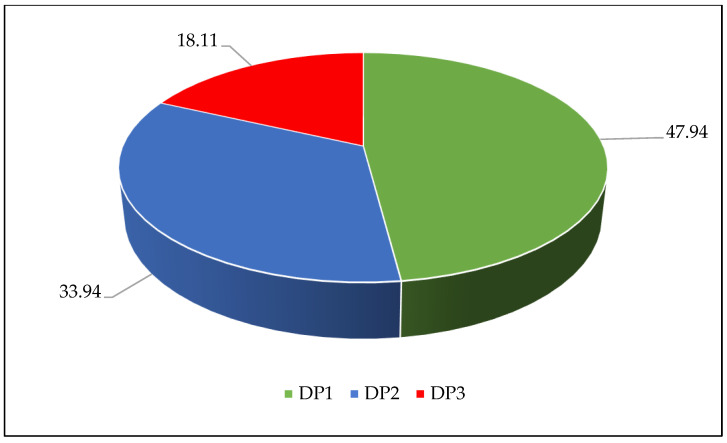
The constitution ratio for each dietary pattern. Abbreviation: DP 1 = “healthy” dietary pattern, DP 2 = “traditional” dietary pattern, DP 3 = “meat/animal protein” dietary pattern (%). Note: Percentages may not add up to 100% due to rounding.

Latent class models with 1–5 classes were estimated, as Table 2 showed. The 3-class solution was selected as the final model for this study because of the lower AIC, BIC, and ssaBIC, higher entropy, and LMR-LRT and BLRT < 0.001 (AIC: 11,462.201; BIC: 11,572.112; ssaBIC: 11,508.573, and entropy: 0.656). Finally, three DPs were detected. Figure 3 presents the estimated class-specific response probabilities, which show the three DPs among the 3-class solution, and the constitution ratio of each DP is illustrated in Figure 4. DP 1 was characterized by individuals with the highest probability of consuming the most food variety, including 47.94% of the samples. Compared to the other DPs, DP 1 can be characterized as a “healthy” DP. DP 2 accounted for one-third of the sample (33.94%). It included those with high probabilities for intake of vegetables–fruits, animal foods such as red meat, aquatic products, and eggs, low possibilities for sufficient milk, and beans–nuts. DP 2 can be described as showing a “traditional” DP. DP 3 was characterized by individuals with a high probability of consuming animal foods such as red meat, aquatic products, and eggs, with low possibilities for sufficient vegetables–fruits, milk, and beans–nuts, including 18.11% of the samples. DP 3 can be labelled as the “meat/animal protein” DP compared to the other DPs.

### 3.4. Distribution of Demographic Information among Participants by the Dietary Diversity and by Dietary Patterns

The distribution of demographic information among participants by the DD is presented in Table 3. Chi-squared tests observed that gender (*χ²* = 16.059, *p* = 0.003), age (*χ*^2^ = 106.496, *p* < 0.001), educational level (*χ*^2^ = 279.110, *p* < 0.001), AHI (*χ*^2^ = 180.460, *p* < 0.001), smoking status (*χ*^2^ = 13.527, *p* = 0.009), obesity (*χ*^2^ = 18.797, *p* < 0.001), and hypertension (*χ*^2^ = 33.835, *p* < 0.001) were significantly associated with the DD. The distribution of demographic information among participants by DPs is presented in Table 4. Chi-squared tests illustrated that gender (*χ*^2^ = 25.793, *p* < 0.001), age (*χ*^2^ = 91.568, *p* < 0.001), educational level (*χ*^2^ = 232.449, *p* < 0.001), AHI (*χ*^2^ = 120.361, *p* < 0.001), smoking status (*χ*^2^ = 21.174, *p* < 0.001), drinking status (*χ*^2^ = 7.098, *p* = 0.029), obesity (*χ*^2^ = 26.836, *p* < 0.001), and hypertension (*χ*^2^ = 36.651, *p* < 0.001) were significantly associated with DPs.

### 3.5. Generalized Linear Regression Analysis of Factors Affecting the Dietary Diversity

As demonstrated in Table 5, generalized linear regression analysis was presented to study the factors associated with DD. Overall, the females (β = −0.23, 95% CI = −0.38 to −0.08, *p* = 0.003) exhibited lower DD compared with the males. According to the educational level group, individuals who had a primary school education (β = 0.23, 95% CI = 0.08 to 0.38, *p* = 0.003), junior high school education (β = 0.61, 95% CI = 0.43 to 0.78, *p* < 0.001), senior high school education (β = 0.92, 95% CI = 0.70 to 1.15, *p* < 0.001), and junior college or above (β = 1.17, 95% CI = 0.93 to 1.41, *p* < 0.001) exhibited higher DD than those below the primary school. Moreover, compared to participants’ AHI of less than 12,000 yuan, those AHI ranging from 20,000 to 59,999 yuan (β = 0.43, 95% CI = 0.24 to 0.62, *p* < 0.001) and 60,000 yuan or more (β = 0.58, 95% CI = 0.37 to 0.80, *p* < 0.001) tended to have higher DD. Additionally, non-obese populations (β = 0.15, 95% CI = 0.02 to 0.29, *p* = 0.020) tended to have higher DD than obese populations.

### 3.6. Multiple Logistic Regression Analysis of Factors Affecting Dietary Patterns

Multiple logistic regression was used to explore the relationship between the demographic factors and three DPs (Table 6). Three DPs were used as dependent variables, and the DP 1 group was used as the reference group. Those factors were used as an independent variable to be added in the multiple logistic regression analysis. The results showed that females (DP 2: OR = 1.45, 95% CI = 1.08 to 1.94, *p* = 0.014, and DP 3: OR = 2.02, 95% CI = 1.39 to 2.94, *p* < 0.001) were more likely to develop DP 2 and DP 3 than males. Participants aged 18–44 years (DP 2: OR = 1.46, 95% CI = 1.02 to 2.10, *p* = 0.039, and DP 3: OR = 0.49, 95% CI = 0.28 to 0.86, *p* = 0.013) were more likely to have DP 2 and less likely to have DP 3, as compared with those who were 60 years or older. However, in terms of educational level, those with a primary school education (DP 2: OR = 0.63, 95% CI = 0.47 to 0.85, *p* = 0.002, and DP 3: OR = 0.65, 95% CI = 0.47 to 0.92, *p* = 0.015), a junior high school education (DP 2: OR = 0.38, 95% CI = 0.27 to 0.53, *p* < 0.001, and DP 3: OR = 0.28, 95% CI = 0.18 to 0.44, *p* < 0.001), a senior high school education (DP 2: OR = 0.22, 95% CI = 0.14 to 0.35, *p* < 0.001, and DP 3: OR = 0.19, 95% CI = 0.10 to 0.35, *p* < 0.001), and a junior college or above (DP 2: OR = 0.13, 95% CI = 0.07 to 0.22, *p* < 0.001, and DP 3: OR = 0.04, 95% CI = 0.01 to 0.14, *p* < 0.001) were less likely to experience DP 2 and DP 3 compared to those with a lower primary school education. When compared with those whose AHI was less than 12,000 yuan, respondents with an AHI ranged from 20,000 to 59,999 yuan (DP 2: OR = 0.69, 95% CI = 0.47 to 1.00, *p* = 0.050, and DP 3: OR = 0.54, 95% CI = 0.35 to 0.82, *p* = 0.005) and over 60,000 yuan (DP 2: OR = 0.45, 95% CI = 0.29 to 0.69, *p* < 0.001, and DP 3: OR = 0.41, 95% CI = 0.24 to 0.68, *p* = 0.001) were less likely to form DP 2 and DP 3. Compared to those obese populations, non-obese populations (OR = 0.59, 95% CI = 0.44 to 0.80, *p* = 0.001) were less likely to establish DP 3. Further, for those without hypertension diseases (OR = 0.71, 95% CI = 0.52 to 0.96, *p* = 0.026), a lower risk was discovered concerning DP 3. Significantly, as for individuals without diabetes disease (OR = 1.50, 95% CI = 1.03 to 2.19, *p* = 0.035), a higher risk was found concerning DP 3.

## 4. Discussion

With significant consequences for public health, diet is a controllable risk factor that should be prioritized [1]. Encouraging a varied diet and a healthy DP could enhance the overall diet since both are equally pivotal links to healthy food and nutrient intake [4,6]. In comparison, rural residents’ DD and DP are often underrepresented in food consumption studies. Understanding DD and DP among rural residents and the related factors to drivers of food choice is essential. It can support informing nutritional guidelines specific to rural resident groups and develop tailored interventions to encourage dietary improvement in the long term. Therefore, this study examined several socio-demographic factors concerning DD and DP in adults from Pingnan, China, and contributed significantly to our understanding of the key measures for enhancing rural residents’ diets. This study adopted six food groups to calculate the total number of food varieties intake to assess DD. We used the method of LCA to determine the DPs in a representative sample and obtain three DPs: “healthy” DP (which included vegetables–fruit, red meat, aquatic products, egg, milk, beans–nuts), “traditional” DP (which included vegetables–fruit, red meat, aquatic products, egg), and “meat/animal protein” DP (which included red meat, aquatic products, egg). According to the study’s findings, gender, educational level, AHI, and obesity were associated with DD; similarly, gender, age, educational level, AHI, obesity, hypertension, and diabetes were the correlated factors for the DPs.

Specifically, females were negatively correlated with the higher DD and more likely to develop the “traditional” DP and “meat/animal protein” DP than males, which is inconsistent with previous studies [18,36]. The survey in Poverty Areas of Northwest China found no significant difference between sex and DD [36]. Additionally, research has shown that females adhere more to healthy DPs than males [17,18]. Although, a study agrees that females in China will learn more dietary knowledge to promote a healthy diet for family members because they are primarily responsible for food preparation [22]. However, compared with males, females in rural areas generally tend to have subordinate status in the household and less access to primarily high-quality food [37]. In addition, no significant relationship between age and DD was found in our analysis, which was not congruent with a former study that showed that the younger aged group had lower DD than the older ones [38]. Interestingly, younger participants in our samples were more likely to have “traditional” DP than older adults, but “meat/animal protein” DP showed the reverse result. This finding is inconsistent with the previous studies showing the same point of view that older adults usually make positive decisions concerning their nutrition and follow healthier DP [19,38]. A possible reason is that, on the one hand, younger people skipped meals more frequently, especially breakfast and night eating, and had fewer servings of dairy products, thus leading to worse DP throughout the day [18]. On the other hand, some older adults living in rural areas limited by the low economic conditions or living alone might spend less money on expensive food like fruits, dairy products, and nuts or purchase less food [16]. Therefore, public health nutrition interventions aiming to enhance dietary knowledge and improve diets should target the group of females, younger people, and older adults.

The findings of this study support a large body of research indicating higher socioeconomic status, including a high level of education and more AHI, substantially correlated with higher DD and healthier DP [9,16,17,20]. Regarding AHI factors, income reflects purchasing power and indicates a person’s financial resources [9]. The determinants of food variety choices are complex, and the price is one of many factors guiding these choices, and a low income can restrain people from spending more money on more food choices [39]. Another often-cited reason for poor DD and DP among low-income individuals is the cost of healthy food [20]. Financially constrained people may consume lower-quality diets, such as fewer fruits and vegetables or more high energy-dense foods, than more affluent populations [20,40]. In terms of educational level, education allows people to obtain information about nutritional knowledge and healthy DP, which ultimately leads to higher DD and better DP. Furthermore, people with a higher educational level tend to have higher incomes and better purchasing power than those with a lower academic level. Therefore, dietary knowledge must be enhanced to improve the poor DD and DP among rural residents with low-education and less-income groups. Meanwhile, the government should step up extensive health education efforts to promote diet-related health education that facilitates the change from unhealthy to healthy eating behaviors in rural areas.

In fact, owing to the complexity of foods and the potential associations between dietary components, the relationship between diet and obesity is intricate [41]. Previous studies have shown a positive association between a higher risk of obesity and DP with increased consumption of red meat abundant in saturated fat and cholesterol [42,43,44]. In the present study, the non-obese population was inversely associated with “meat/animal protein” DP, indicating that rural residents with obesity who consume more meat or animal food deserve further attention. Nevertheless, we found a positive relationship between the non-obese population and higher DD, which was inconsistent with prior finds in the literature [45,46]. A study in a less developed region suggested that obesity was associated with higher DD among adults in southwest China [45]. One explanation may be that rural residents in the non-obese population tend to increase their intake of healthy foods, such as fruit and vegetables, rather than meat, though, it has been regarded that higher DD is associated with higher consumption of total energy and is linked to obesity [46], whereas a systematic review showed that the relationship between adiposity and DD depends on the healthy degree of eatables and variety of all kinds of food, and a healthy diet implies a reduction in metabolic-related risks [47]. For example, a lower risk of obesity was associated with a low intake of healthy foods (fruits and vegetables) that contributes to higher DD [48]. Another explanation for the finding might be that a varied but balanced diet is attributed to providing a rational nutrient supply. The literature suggests that one reason for increasing obesity is the dietary imbalance coming with DD [45]. Briefly, those who have obesity should be advocating eating healthy foods (e.g., fruits and vegetables) and a balanced diet in rural areas.

According to the results of a systematic review and a dose–response meta-analysis between food groups and the risk of hypertension, it is generally accepted that an increased risk of hypertension was associated with red meat and processed meat [49]. Based on the findings of the present study, those participants without hypertension were negatively associated with “meat/animal protein” DP, which underscores a healthy diet is essential for participants with hypertension in rural areas. Interestingly, our study showed that those participants without diabetes were positively associated with “meat/animal protein” DP, which confirms the relationship between diabetes and diet factors. People with diabetes are likely to follow a healthier diet than those without diabetes possibly because they have learned about nutrition knowledge from physicians and followed a diabetic diet for glycemic control [50]. This suggests that people without diabetes should also be taken into consideration to increase their ability to choose healthier diets in rural areas where nutrition and health knowledge are inadequate. Notably, we did not observe an association between smoking or drinking and DD or DP, which is inconsistent with many previous studies [4,46,51]. A possible reason for this finding is that the behavior factors (e.g., smoking and drinking) might have a smaller impact on the dietary choices of the rural residents than other factors. Previously published studies have demonstrated the clustering of health/risk behaviors [52,53,54]. Additionally, evidence shows that changes in one risk behavior are related to changes in another behavior [54]. Thus, the association between smoking or drinking and diet in the Pingnan region needs further study.

A strength of our study was that it included a rigorous sampling process and investigation process, which used multistage systematic clustered random sampling, the use of validated tools for assessing diet, and conducted quite strict quality control to obtain high-quality, representative data to increase the validity and generalizability of our findings. Further, this study used the LCA method to identify DPs and provides a new perspective. However, several limitations of this study should be noted. First, the cross-sectional study design allows us to describe associations but it was difficult to determine causality or explore the direction of associations based on the present findings. Second, the estimation of food intake was based on retrospective self-reports from the past 12 months using the FFQ. Although well-trained investigators conducted interviews to help improve accuracy, recall bias may still result in overestimating or underestimating intake. Third, we focused on analyzing socio-demographic factors but lacked other potential variables, as many factors may influence food intake. Thus, more research focusing on rural residents must include additional elements for a comprehensive assessment.

## 5. Conclusions

In conclusion, the present study identified DD and DP socio-demographic factors in Pingnan, China. Females exhibited lower DD and were more likely to adhere to “traditional” DP and “meat/animal protein” DP. Those with higher educational levels and AHI were positively associated with higher DD, while less likely to have “traditional” DP and “meat/animal protein” DP. Non-obese people exhibited higher DD and were less likely to have “meat/animal protein” DP. Our study suggests that vulnerable populations which tend to have a lower DD and are more likely to adhere to “traditional” DP and “meat/animal protein” DP, which is most evident among those who are female, those with lower educational levels and AHI, and obese people. Our findings highlight that the policymakers must perform specific diet-related health promotion measures and interventions that target these vulnerable populations to improve a healthier DD and DP. For instance, implementing health interventions, public education, and support programs for rural communities, particularly promoting greater diversity and a wider variety of food intake as well as eating healthy foods (such as fruits and vegetables) and balanced diets.

## Figures and Tables

**Table 1:** Demographic information for survey participants (n = 1800).

Variable		Participants	Constitutional Ratio (%)
Gender	Male	888	49.33
	Female	912	50.67
Age (years)	18–44	335	18.61
	45–59	746	41.44
	≥60	719	39.95
Marital status	Married	1556	86.44
	Others	244	13.56
Educational level	Below primary school	534	29.66
	Primary school	529	29.39
	Junior high school	380	21.11
	Senior high school	194	10.78
	Junior college or above	163	9.06
AHI (CNY)	<12,000	234	13.00
	12,000–19,999	352	19.56
	20,000–59,999	785	43.61
	≥60,000	429	23.83
Smoking	Yes	523	29.06
	No	1277	70.94
Drinking	Yes	495	27.50
	No	1305	72.50
Obesity	Yes	497	27.61
	No	1303	72.39
Hypertension	Yes	610	33.89
	No	1190	66.11
Diabetes	Yes	311	17.28
	No	1489	82.72

Abbreviation: AHI = annual household income; CNY = China Yuan.

**Table 2:** Latent class analysis model fit statistics.

Model	AIC	BIC	ssaBIC	LRT *p*-Value	BLRT *p*-Value	Entropy
1-class	12,367.612	12,400.585	12,381.524	-	-	-
2-class	11,525.404	11,596.846	11,555.546	<0.001	<0.001	0.633
3-class	11,462.201	11,572.112	11,508.573	<0.001	<0.001	0.656
4-class	11,438.537	11,586.916	11,501.139	0.004	<0.001	0.586
5-class	11,440.136	11,626.984	11,518.968	0.182	1.000	0.622

Abbreviation: AIC = Akaike Information Criterion, BIC = Bayesian Information Criterion, ssaBIC = sample size adjusted Bayesian Information Criterion, LRT = Lo–Mendell–Rubin likelihood ratio test, BLRT = Bootstrapped Likelihood Ratio Test.

**Table 3:** Distribution of demographic information among participants by the dietary diversity.

Variable	DD ≤ 2 (n = 330)	DD = 3 (n = 340)	DD = 4 (n = 414)	DD = 5 (n = 425)	DD = 6 (n = 291)
Gender (*χ*^2^ = 16.059, *p* = 0.003)					
Male	170 (19.14)	175 (19.71)	227 (25.56)	197 (22.18)	119 (13.40)
Female	160 (17.54)	165 (18.09)	187 (20.50)	228 (25.00)	172 (18.86)
Age (years) (*χ*^2^ = 106.496, *p* < 0.001)					
18–44	28 (8.36)	51 (15.22)	75 (22.39)	85 (25.37)	96 (28.66)
45–59	109 (14.61)	148 (19.84)	181 (24.26)	187 (25.07)	121 (16.22)
≥60	193 (26.84)	141 (19.61)	158 (21.97)	153 (21.28)	74 (10.29)
Marital status (*χ*^2^ = 6.135, *p* = 0.189)					
Married	281 (18.06)	295 (18.96)	346 (22.24)	377 (24.23)	257 (16.52)
Others	49 (20.08)	45 (18.44)	68 (27.87)	48 (19.67)	34 (13.93)
Educational level (*χ*^2^ = 279.110, *p* < 0.001)					
Below primary school	158 (29.59)	122 (22.85)	131 (24.53)	81 (15.17)	42 (7.87)
Primary school	116 (21.93)	118 (22.31)	127 (24.01)	104 (19.66)	64 (12.10)
Junior high school	43 (11.32)	66 (17.37)	97 (25.53)	109 (28.68)	65 (17.11)
Senior high school	11 (5.67)	26 (13.40)	31 (15.98)	67 (34.54)	59 (30.41)
Junior college or above	2 (1.23)	8 (4.91)	28 (17.18)	64 (39.26)	61 (37.42)
AHI (CNY) (*χ*^2^ = 180.460, *p* < 0.001)					
<12,000	82 (35.04)	62 (26.50)	44 (18.80)	35 (14.96)	11 (4.70)
12,000–19,999	92 (26.14)	69 (19.60)	94 (26.70)	70 (19.89)	27 (7.67)
20,000–59,999	122 (15.54)	152 (19.36)	183 (23.31)	193 (24.59)	135 (17.20)
≥60,000	34 (7.93)	57 (13.29)	93 (21.68)	127 (29.60)	118 (27.51)
Smoking (*χ*^2^ = 13.527, *p* = 0.009)					
Yes	105 (20.08)	112 (21.41)	128 (24.47)	116 (22.18)	62 (11.85)
No	225 (17.62)	228 (17.85)	286 (22.40)	309 (24.20)	229 (17.93)
Drinking (*χ*^2^ = 3.392, *p* = 0.495)					
Yes	90 (18.39)	97 (18.62)	107 (23.52)	110 (24.14)	91 (15.33)
No	240 (18.18)	243 (19.60)	307 (21.62)	315 (22.22)	200 (18.38)
Obesity (*χ*^2^ = 18.797, *p* < 0.001)					
Yes	119 (23.94)	100 (20.12)	106 (21.33)	108 (21.73)	64 (12.88)
No	211 (16.19)	240 (18.42)	308 (23.64)	317 (24.33)	227 (17.42)
Hypertension (*χ*^2^ = 33.835, *p* < 0.001)					
Yes	148 (24.26)	119 (19.51)	146 (23.93)	127 (20.82)	70 (11.48)
No	182 (15.29)	221 (18.57)	268 (22.52)	298 (25.04)	221 (18.57)
Diabetes (*χ*^2^ = 4.754, *p* = 0.314)					
Yes	63 (20.26)	67 (21.54)	66 (21.22)	74 (23.79)	41 (13.18)
No	267 (17.93)	273 (18.33)	348 (23.37)	351 (23.57)	250 (16.79)

Abbreviation: AHI = annual household income; CNY = China Yuan. DD = dietary diversity.

**Table 4:** Distribution of demographic information among participants by three dietary patterns.

Variable	DP 1 (n = 863)	DP 2 (n = 611)	DP 3 (n = 326)
Gender (*χ*^2^ = 25.793, *p* < 0.001)			
Male	387 (43.58)	301 (33.90)	200 (22.52)
Female	476 (52.19)	310 (33.99)	126 (13.82)
Age (years) (*χ*^2^ = 91.568, *p* < 0.001)			
18–44	212 (63.28)	103 (30.75)	20 (5.97)
45–59	371 (49.73)	262 (35.12)	113 (15.15)
≥60	280 (38.94)	246 (34.21)	193 (26.84)
Marital status (*χ*^2^ = 0.851, *p* = 0.653)			
Married	751 (45.90)	528 (34.02)	277 (20.08)
Others	112 (48.26)	83 (33.93)	49 (17.80)
Educational level (*χ*^2^ = 232.449, *p* < 0.001)			
Below primary school	161 (30.15)	229 (42.88)	144 (26.97)
Primary school	214 (40.45)	196 (37.05)	119 (22.50)
Junior high school	211 (55.53)	124 (32.63)	45 (11.84)
Senior high school	138 (71.13)	41 (21.13)	15 (7.73)
Junior college or above	139 (85.28)	21 (12.88)	3 (1.84)
AHI (CNY) (*χ*^2^ = 120.361, *p* < 0.001)			
<12,000	68 (29.06)	93 (39.74)	73 (31.20)
12,000–19,999	126 (35.80)	149 (42.33)	77 (21.88)
20,000–59,999	386 (49.17)	270 (34.40)	129 (16.43)
≥60,000	283 (65.97)	99 (23.08)	47 (10.96)
Smoking (*χ*^2^ = 21.174, *p* < 0.001)			
Yes	219 (41.87)	177 (33.84)	127 (24.28)
No	644 (50.43)	434 (33.99)	199 (15.58)
Drinking (*χ*^2^ = 7.098, *p* = 0.029)			
Yes	234 (47.27)	153 (30.91)	108 (21.82)
No	629 (48.20)	458 (35.10)	218 (16.70)
Obesity (*χ*^2^ = 26.836, *p* < 0.001)			
Yes	195 (39.24)	181 (36.42)	121 (24.35)
No	668 (51.27)	430 (33.00)	205 (15.73)
Hypertension (*χ*^2^ = 36.651, *p* < 0.001)			
Yes	247 (40.49)	209 (34.26)	154 (25.25)
No	616 (51.76)	402 (33.78)	172 (14.45)
Diabetes (*χ*^2^ = 1.895, *p* = 0.388)			
Yes	141 (45.34)	116 (37.30)	66 (21.22)
No	722 (48.49)	495 (33.24)	348 (23.37)

Abbreviation: AHI = annual household income; CNY = China Yuan. DP 1 = “healthy” dietary pattern, DP 2 = “traditional” dietary pattern, DP 3 = “meat/animal protein” dietary pattern.

**Table 5:** Generalized linear regression analysis of factors associated with the dietary diversity.

Variable	β (95%CI)	*p*
Gender (reference = Male)	-	-
Female	−0.23 (−0.38, −0.08)	0.003
Age (years) (reference = ≥60)	-	-
18–44	0.08 (−0.11, 0.26)	0.423
45–59	0.04 (−0.10, 0.17)	0.612
Marital status (reference = Married)	-	-
Others	0.04 (−0.13, 0.21)	0.641
Educational level (reference = Below primary school)	-	-
Primary school	0.23 (0.08, 0.38)	0.003
Junior high school	0.61 (0.43, 0.78)	<0.001
Senior high school	0.92 (0.70, 1.15)	<0.001
Junior college or above	1.17 (0.93, 1.41)	<0.001
AHI (CNY) (reference = <12,000)	-	-
12,000–19,999	0.15 (−0.05, 0.36)	0.149
20,000–59,999	0.43 (0.24, 0.62)	<0.001
≥60,000	0.58 (0.37, 0.80)	<0.001
Smoking (reference = Yes)	-	-
No	0.09 (−0.07, 0.25)	0.274
Drinking (reference = Yes)	-	-
No	0.04 (−0.11, 0.18)	0.608
Obesity (reference = Yes)	-	-
No	0.15 (0.02, 0.29)	0.020
Hypertension (reference = Yes)	-	-
No	0.12 (−0.01, 0.25)	0.073
Diabetes (reference = Yes)	-	-
No	−0.08 (−0.23, 0.08)	0.317

Abbreviation: AHI = annual household income; CNY = China Yuan.

**Table 6:** Multiple logistic regression analysis comparing dietary pattern 2 and 3 to dietary pattern 1.

Variable	DP 2		DP 3	
	OR (95%CI)	*p*	OR (95%CI)	*p*
Gender (reference = Male)	-	-	-	-
Female	1.45 (1.08, 1.94)	0.014	2.02 (1.39, 2.94)	<0.001
Age (years) (reference = ≥60)	-	-	-	-
18–44	1.46 (1.02, 2.10)	0.039	0.49 (0.28, 0.86)	0.013
45–59	1.29 (0.98, 1.68)	0.065	0.77 (0.56, 1.07)	0.119
Marital status (reference = Married)	-	-	-	-
Others	0.91 (0.65, 1.27)	0.572	0.86 (0.57, 1.30)	0.474
Educational level (reference = Below primary school)	-	-	-	-
Primary school	0.63 (0.47, 0.85)	0.002	0.65 (0.47, 0.92)	0.015
Junior high school	0.38 (0.27, 0.53)	<0.001	0.28 (0.18, 0.44)	<0.001
Senior high school	0.22 (0.14, 0.35)	<0.001	0.19 (0.10, 0.35)	<0.001
Junior college or above	0.13 (0.07, 0.22)	<0.001	0.04 (0.01, 0.14)	<0.001
AHI (CNY) (reference = <12,000)	-	-	-	-
12,000–19,999	1.00 (0.67, 1.51)	0.990	0.78 (0.49, 1.25)	0.310
20,000–59,999	0.69 (0.47, 1.00)	0.050	0.54 (0.35, 0.82)	0.005
≥60,000	0.45 (0.29, 0.69)	<0.001	0.41 (0.24, 0.68)	0.001
Smoking (reference = Yes)	-	-	-	-
No	0.99 (0.72, 1.38)	0.976	0.84 (0.57, 1.23)	0.374
Drinking (reference = Yes)	-	-	-	-
No	1.05 (0.79, 1.40)	0.720	0.77 (0.55, 1.08)	0.130
Obesity (reference = Yes)	-	-	-	-
No	0.80 (0.62, 1.04)	0.095	0.59 (0.44, 0.80)	0.001
Hypertension (reference = Yes)	-	-	-	-
No	0.97 (0.75, 1.26)	0.842	0.71 (0.52, 0.96)	0.026
Diabetes (reference = Yes)	-	-	-	-
No	0.97 (0.72, 1.31)	0.861	1.50 (1.03, 2.19)	0.035

Abbreviation: AHI = annual household income; CNY = China Yuan. DP 2 = “traditional” dietary pattern, DP 3 = “meat/animal protein” dietary pattern.

## Data Availability

The data are not publicly available due to the data containing information that could compromise the participants’ privacy.
